# “Hybrid” scientific conference: lessons learned from the digital annual meeting of the CARS international conference during the Covid-19 pandemic

**DOI:** 10.1515/iss-2021-0012

**Published:** 2021-11-09

**Authors:** Daniel Ostler, Jana Steger, Lukas Bernhard, Kevin Yu, Regine Hartwig, Hubertus Feussner, Dirk Wilhelm

**Affiliations:** Minimally Invasive Interdisciplinary Therapeutical Intervention, Klinikum rechts der Isar, Technical University Munich, Munich, Germany; Department of Surgery, Klinikum rechts der Isar, Technical University Munich, Munich, Germany

**Keywords:** Covid-19 pandemic, digital conferences, feasibility of digital communication, scientific communication

## Abstract

**Objectives:**

Due to the coronavirus disease 2019 (Covid-19) pandemic, all scientific conferences in the year 2020 had to be adapted in their form of presence to accommodate for safety regulations, postponed, or canceled entirely. As organizers of the annual Computer Assisted Radiology & Surgery International Conference & Exhibition (CARS)-Conference 2020, we decided to hold a “hybrid” conference, i.e., a virtual conference with partial presence to mitigate the drawbacks of a purely virtual conference. It is the purpose of this paper to describe the results and experience gained by our first hybrid conference.

**Methods:**

Besides technical necessities like an online conferencing tool, we introduced additional personal namely the technical chairs and communication officers ensuring a smooth flow of presentations. To measure the success of the hybrid conference, we assessed various parameters during the conference (e.g., counting of adverse events, delays, and no-shows) and sent a questionnaire to participants for evaluation after the conference.

**Results:**

We offered four types of presentation formats, whereas the majority of speakers presented their pre-produced videos including live discussions. Significant delays in sessions occurred during the morning sessions, which could be reduced during lunch breaks. The analysis of the influence of the distribution of the audience’s location/time zone toward the attendance rate showed a high relevance for the American zone and only little influence for the Asian-Pacific region. Based on the questionnaire, 60% of responders considered the hybrid approach as superior and 12% as inferior to purely virtual conferences.

**Conclusions:**

Most scientific associations in 2020 had to struggle with a dramatic change: Regular, traditional meetings with personal communication and exchange, networking, and creation of new visions became obsolete almost instantly. As an alternative, virtual conferences became increasingly popular, and are offering additional advantages (e.g., reduction of cost for travel, lodging, and time on transit). To overcome the drawbacks of purely virtual conferences, we introduced a hybrid concept for the CARS-Congress. While certainly, those with the privilege to take part personally on-site did benefit most from the hybrid format. Facing upcoming waves of the Covid-19 Pandemic, with ongoing changes to the regulations on meetings and transit, hybrid conferences are a viable option for scientific conferences for the future.

## Introduction

The first wave of coronavirus disease 2019 (Covid-19) pandemic struck human civilization worldwide to an extent which was completely unexpected. Public life was paralyzed overnight since severe restrictions of mobility and other activities were enacted.

Self evidently, scientific meetings had to be canceled immediately as well. As an alternative to complete cancellation, online- or virtual reality meetings were often established. Online conferences were, of course, already performed long before the Covid-19 disruption [1], but now a really broad use was observed. However, one of the essential components of a conference – the real-time social interaction among the participants – is, thus, significantly reduced. With the rapid oncome of the pandemic in Germany in spring 2020, it became immediately clear that the annual meeting of Computer Assisted Radiology & Surgery International Conference & Exhibition (CARS) which was scheduled for June, 23rd–26th, was highly improbable to happen as it had initially been planned. The classical format of the CARS annual conference, well established over the years, was a multitrack conference with some common events and a rich social program fostering direct communication among all participants. Insisting on this classical format would have required postponing the annual meeting to a later time (with severe difficulties to define a realistic date) or canceling it completely for the year 2020. None of it seemed to be an option for us. Accordingly, a virtual conference had to be considered.

When we decided to prepare an online conference, we had an intensive debate upon how to mitigate or even eliminate the well-known drawbacks of purely virtual conferences without compromising the safety regulation of the Covid-19 precautions. The idea was to initiate a so-called “hybrid meeting” – a conference which consists of a local core of in-person and many virtual attendants from all over the globe who meet in one lecture hall and who also can actively take part as speakers, chairpersons, or debaters [2]. By creating a visual/spatial and temporal anchor, we tried to induce a feeling of “being together” and to avoid the “lost in space” atmosphere or even cybersickness [3], which often arises under the very abstract conditions of the purely virtual conference, in particular, if they last for more than just one day. It is the purpose of this paper to describe the results and experience gained by our first hybrid conference.

## Materials and methods

All members of CARS were informed via e-mail about this new format of CARS 2020. To achieve maximum stability of the final program, we summoned up all applicants to send in pre-produced video recordings as a backup in case of telecommunication problems during the conference. After a careful review, the selected papers and corresponding videos were integrated into the program.

### Hardware, software, and connectivity

As a live casting/conferencing service, a Zoom Webinar 500 License (Zoom Video Communication, San José, CA, USA) was used. Hereby only panelists (Chairpersons and presenting authors) have the rights to share audio/video. Regular attendees are restricted to “view-only” rights, and hence can watch the session and engage via a Q&A-Chat or raise their hand. However, unmuting or showing their webcam is only possible after permission from the host.

Within the physical conference room in Munich, a Cisco SX80 Telepresence Codec (Cisco Systems, Inc. San José, CA, USA) with two pan-tilt-zoom cameras was used. One camera was showing the speaker and/or the local chairpersons, while the other camera was used to show the auditorium, e.g., in case of questions from local in-person attendees.

For the broadcasting of the authors’ videos, Zoom’s screen sharing option with optimization for video playback was used on one of the host personal computers (PC).

### Types of presentation

Four different modes of presentation were conceivable:

If the speaker was physically present on the site of the conference, he/she could give the presentation conventionally. Remote speakers could also present their slides/videos live during their presentation from outside and, then, were available for the online discussion. Another version is to present the pre-produced video including the spoken comment, with the author attending passively. After the presentation, she/he is ready for the live discussion. If the author was not present at all (either in-house or remotely), his/her contribution had to be limited to the pre-produced video alone. In these instances, a direct discussion with the author was not possible.

The different options are delineated in Table 1.

**Table 1:** Types of different talk formats.

A	Personal talk on-site with ad hoc presentation of slides and videos including discussion
B	Remote talk with ad hoc presentation of slides and videos including discussion
C	Display of pre-produced videos/slides with virtual attendance of the speaker and live discussion
D	Display of the pre-produced video without the author

According to the local regulations, the number of persons on-site was restricted to 50 at a time. Members of the Technical University of Munich were admitted only. A strict policy of hygienic precautions was mandatory (a personal distance of 150 cm, disinfection, regular breaks for airing, etc.)

### Personal during the conference

As in any conference, a team of skilled personnel with various roles and responsibilities is key to the success of the conference.

#### (Co-) chairs

The chairpersons (usually 2–3 per session) are the key persons in conventional scientific meetings. They are the managers of their sessions. Accordingly, they should possess a superior understanding of the scientific topics of every single session, sufficient organizational capabilities to keep the sequence of the talks within the time limit, and to ensure an adequate time for discussion. They usually coordinate their actions in a direct dialog between the co-moderators, either a while before or latest at the beginning of the respective session.

#### Technical chair

Since we assumed that a direct (internal) interaction between the chairs is difficult to establish during the video conference (VC) sessions, and the flexible response to any problems occurring throughout the sessions is very limited to a remote chair, we created the role/function of an (additional) Technical Chair (TC). Her/his mission was designed to support the chairs in any technical and organizational regard enabling them to manage the talks and debates as required. In the worst case, they should even be capable to moderate the session alone.

All TC’s were instructed previously about their tasks and obligations:

– To check all videos of their session to gain a good overview.
– To inform themselves about the speakers and their working team.
– To prepare questions for discussions, in case of the absence of the co-chair.
– To get into contact with their co-chairs in due time before the conference to introduce themselves as TC and to explain their role and tasks.
– To start the online session, and perform a last technical system check 15 min before the scheduled start time together with the panelists and co-chairs.

During the session, the TC had to give the starting signal to enable the regular chairs to begin the moderation. Thus, the waiting of one on the other should be eliminated. Each TC was assisted by a so-called communication officer (CO). The CO’s (technically qualified students of engineering or computer science) supported the TC in any technical and organizational regard.

### Defining a physical reference point

Necessarily, a worldwide reference time had to be defined. We chose Central European Summer Time (CEST).

**Figure 1:**
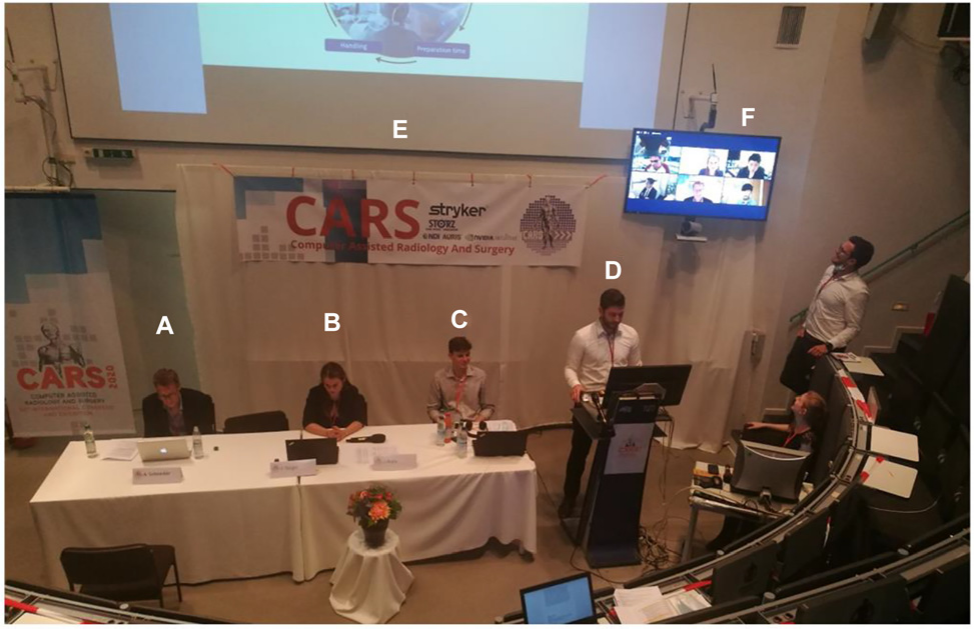
A look into the lecture hall: (A) regular chairperson, present in-person, (B) technical chair, (C) communication officer, (D) speaker, delivering his presentation in-person (Type A), (E) main screen with the slides/videos of the presenter, and (F) auxiliary screen with the remote chairperson and the other speakers of the session. Note the banners and drapes signaling the brand of the event.

In addition to this anchor in temporal regards, we assumed that a spatial fix point would also be helpful. We defined a lecture hall situated in the University hospital of Technische Universität, Munich/Germany as the central venue. The CARS-specific decoration was provided to facilitate identification. Remote visitors/guests should get familiar with the core venue (Figure 1).

### Measures to alleviate the global time difference

Roughly, three time zones: (Zone 1) Europe/Western Asia/Africa; (Zone 2) North and South America; and (Zone 3) Eastern Asia/Australia were covered. With the start at 8:00 CEST in Munich, it was about midnight for the participants living in zone 2 and about 4 PM for those in zone 3. At the end of the program at 18:00 CEST, guests from zone 2 were in the middle of their working hours, whereas it was late in the night in zone 3. As a logical consequence, the presentations of zone 3 should be positioned predominantly in the morning sessions and the contributions from zone 2 in the afternoon sessions.

### Assessment of performance

#### Incidence and number of adverse events

Telecommunication always encompasses the risk of various technical malfunctions, reaching from the maximum credible accident of a complete breakdown of the streaming to minor irritating effects such as a slight transmission delay or faulty illumination. The TC’s and the CO’s were asked to document all types of malfunctions and irregularities.

#### Starting and changing times

In every single case, the time between the official beginning of the individual session (as indicated in the program) and the actual beginning (opening of the session by one of the chairpersons) was accurately documented.

The time required between the upcall of the presentation and the actual start of it (changing time) should be as short as possible. In classical conferences, the former speaker has to leave the speaker’s desk, whereas the next one approaches, positions himself at the lectern and, then, starts the new presentation. In the case of telepresented lectures, the changing time could theoretically be shorter. The exact measurement of changing times was also the task of the CO’s.

#### Retrospective views of the TC’s

All Technical Chairs were interviewed upon their general experiences gained before and throughout the congress using a standardized questionnaire. They were asked to what extent they really could meet the tasks as mentioned above. Moreover, a self-evaluation of their role and function (including the CO’s) was requested.

##### Appraisement by the participants

Any scientific meeting aims to provide an added value to the participant. Accordingly, the organizers were highly interested in gaining information about the view of those who did actively or passively took part in this hybrid conference. Since little is known up to now about the acceptance of hybrid conferences by the international audience, a post hoc survey was organized. All of them were asked about their previous experiences with virtual conferences, their opinion on the hybrid format including the function and performance of TC’s and CO’s, and how much they did feel “at home” in the hybrid conference.

## Results

**Figure 2:**
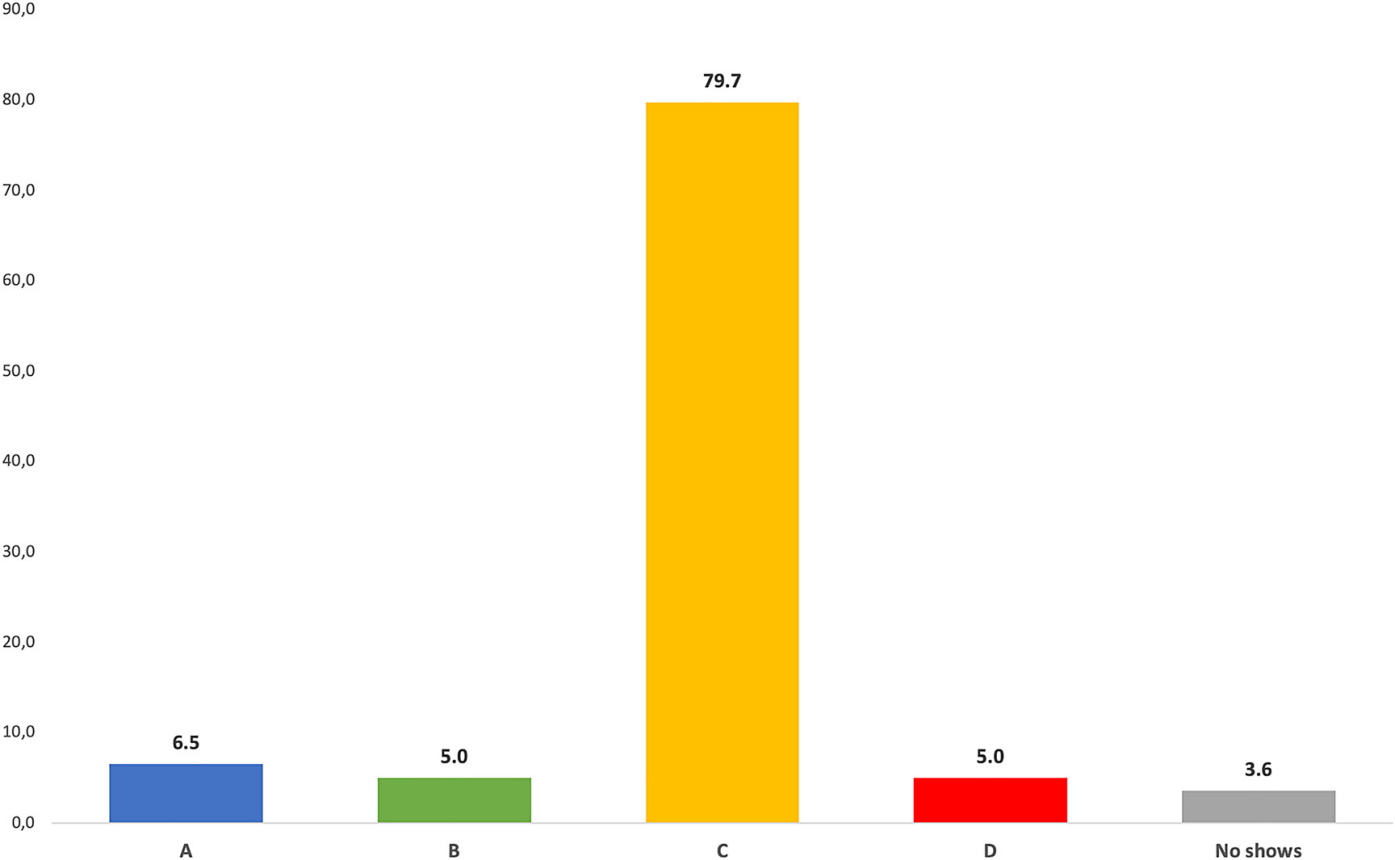
Distribution of types of presentation: (A) on-site oral presentation, (B) remote talk with ad hoc presentation of slides and videos including discussion, (C) display of pre-produced videos/slides with virtual attendance of the speaker and live discussion, and (D) display of the pre-produced video without the author. The majority of speakers presented their pre-produced videos including the live discussion with the virtual audience.

As mentioned above, we had summoned up all speakers to send us a pre-produced video in due time before the conference. The overwhelming majority of the speakers responded to the recommendation (Figure 2).

### No shows

In conventional congresses, it happens that speakers do not show if their talk is called up by the chair. This is usually considered annoying and disappointing for the audience and chairs, but sometimes helpful in the case that the session has run overtime. Depending on the type, venue, and character of the meeting, the rate of no-shows may be as high as 10–15% (this value is derived from personal experience since it is highly dependent on the type of the conference; only limited data is available in literature as e.g., in Ref. [4]). In our instance, four cases were observed (2%).

### Start of the sessions

**Figure 3:**
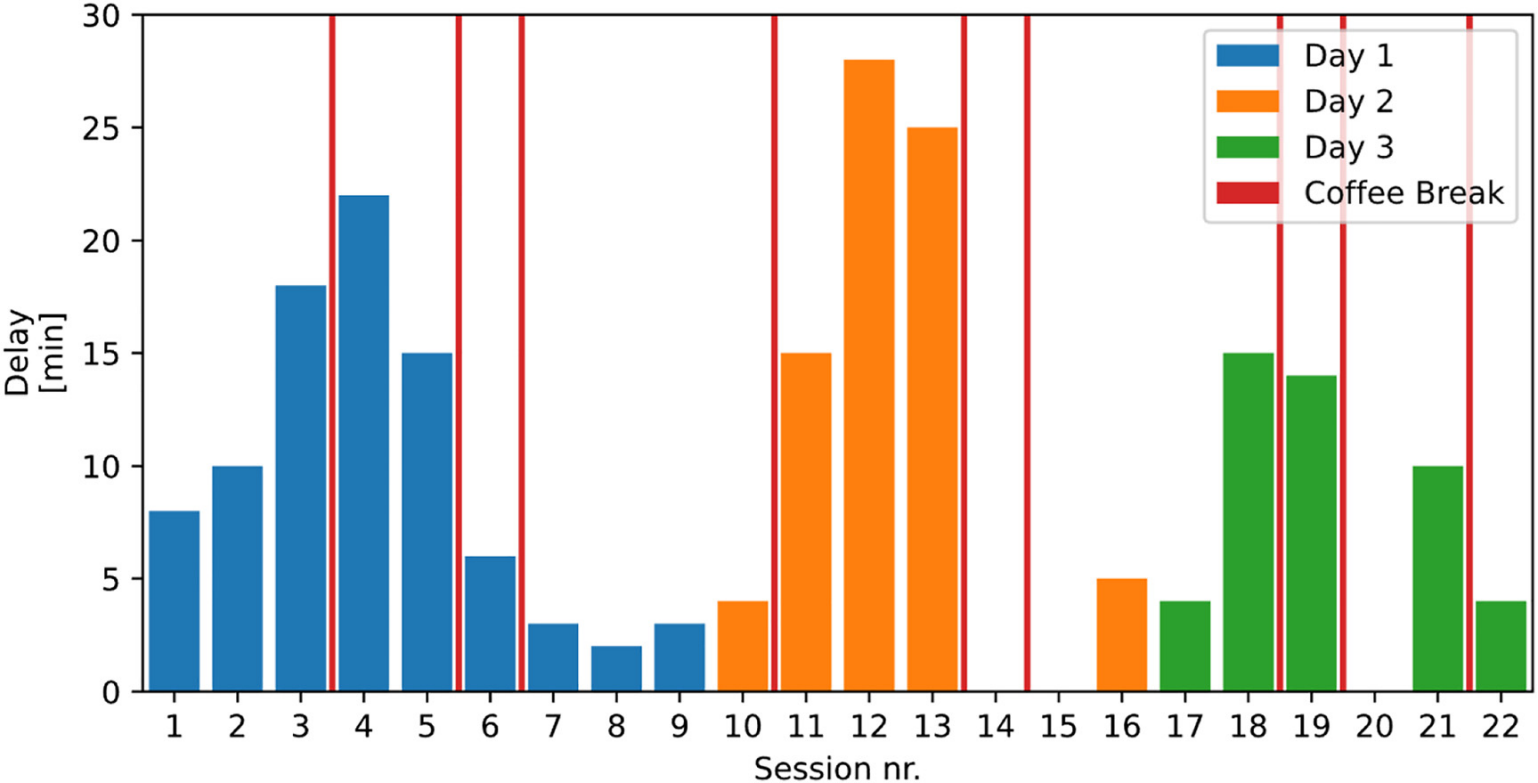
Temporal difference between scheduled session opening time (program) and actual beginning (blue: day 1, orange: day 2, green: day 3).

Although the time schedule seemed to be fitting when the program had been established, some considerable delays did already soon occur as shown in Figure 3. It has to be emphasized, however, that this was not due to technical reasons.

The average duration of all pre-produced videos was 10±2.5 min. The coffee breaks in the morning (15 min) did not help much to abbreviate the delay, as opposed to the lunch break (75 min) and the afternoon coffee breaks (15 min) which could be used to compensate for overtime. The small number of live on-site presentations did not have a relevant influence on the prolongation.

### Time to switch from one presentation to the next (changing time)

**Figure 4:**
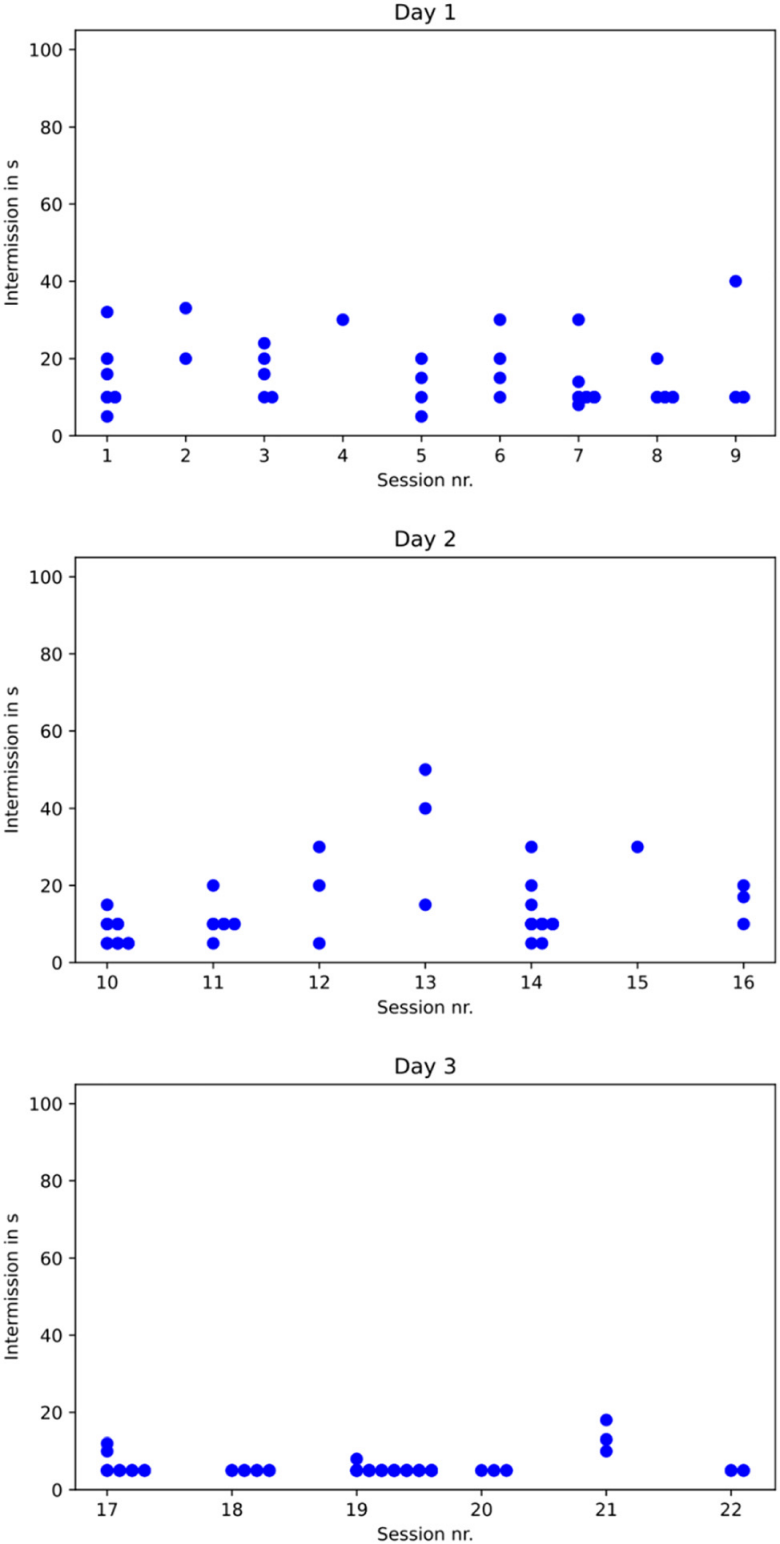
Length of the intervals (in seconds) between the individual presentations per session.

Although the TC’s and their communication officers always tried to stand on the ready to switch to the successive presentation, the time between the end of the preceding presentation and the beginning of the next varied considerably (Figure 4).

#### Incidence and type of irregularities

Severe irregularities or failures did not occur. Audio feedback and background noise were the most frequent problems, but could always be eliminated with a few seconds to minutes. Loss of sound for several minutes occurred in three instances, whereas frozen image impaired the presentation in five sessions. Fortunately, a complete breakdown of the transmission was avoided. From the technical side, the conditions were always comparatively stable and reliable.

#### Impact of the global time difference

**Figure 5:**
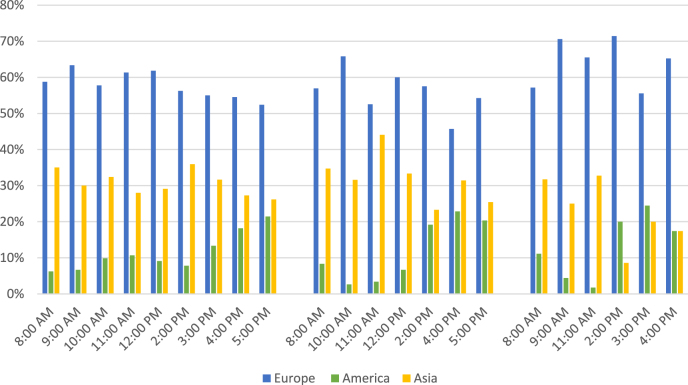
Participants per time zone/time of the day: As expected, the percentage of zone 1 (Europe) was rather stable. Participants of time zone 2 (America) preferred the CEST afternoon or evening, whereas the number of guests from time zone 3 (Asia) did not significantly decrease if it was very late in the night in their time zones.

Since it was not only possible to assess the number of participants on average but also the spatial and temporal distribution, some conclusions can be drawn concerning the influence of local time on the attendance of the international audience (see Figure 5).

Some remarkable observations were made. The percentage of participants of time zone 1 (Europe) was rather stable all over the day, which is little wonder since the program was based upon their normal working hours. In time zone 2 (America), participation increased clearly with the beginning of the normal working hours. On the other hand, guests from time zone 3 (Asia) remained present even when it was very late in their respective countries.

### The role of the technical chairs and the communication officers

**Figure 6:**
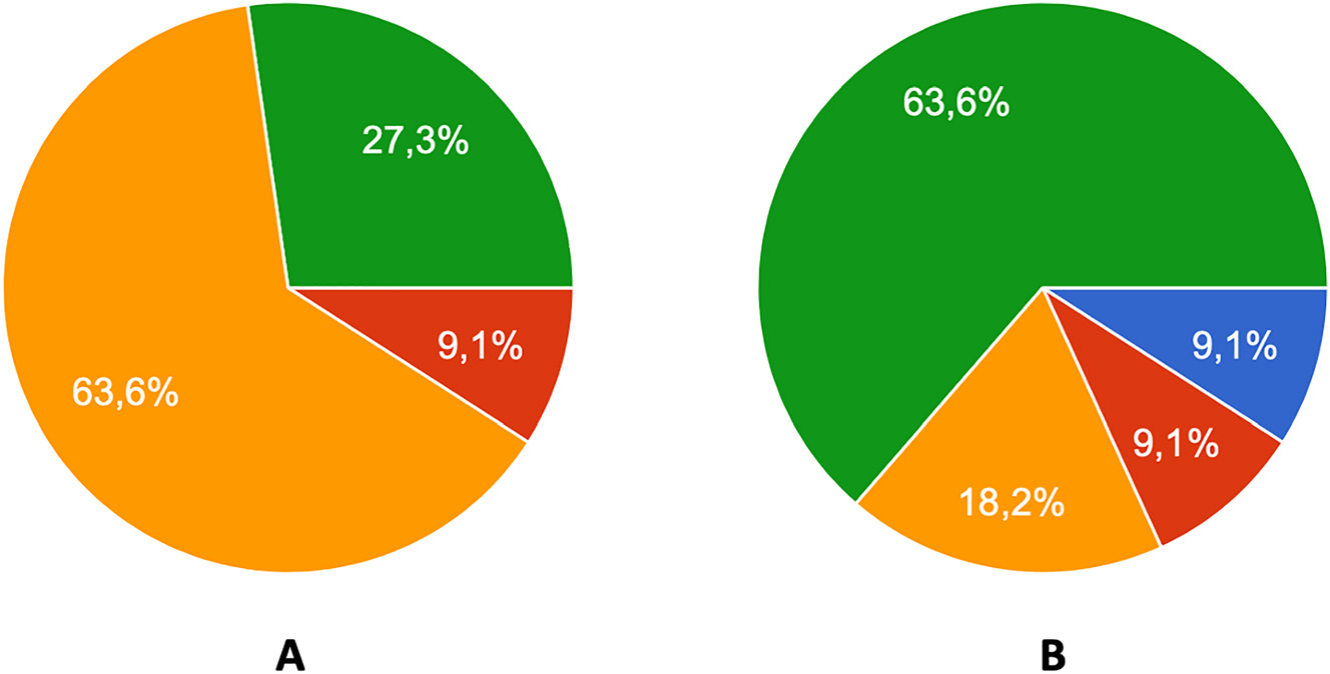
Interview results of TCs regarding the preparation of their session. (A) Percentage of speakers who could be contacted beforehand, (B) percentage of reviewed videos (blue: 0–30%, red: 30–60%, orange: 60–90%, and green: all).

The preparative efforts of the TC’s before the conference were considerable. Based on a structured interview of n=11 TC’s, the overall activities could be described in detail. The technical chairs succeeded to contact all chairpersons before the congress to coordinate the individual sessions, mostly by mail but in a third of cases via videoconference. The TC’s even managed to liaise with most of the individual speakers (Figure 6A) and they were able to review the pre-recorded videos in the majority of cases beforehand (Figure 6B).

During the sessions, they mainly had organizational tasks, since technical issues were primarily dealt with by the communication officers.

### The response of the audience

**Figure 7:**
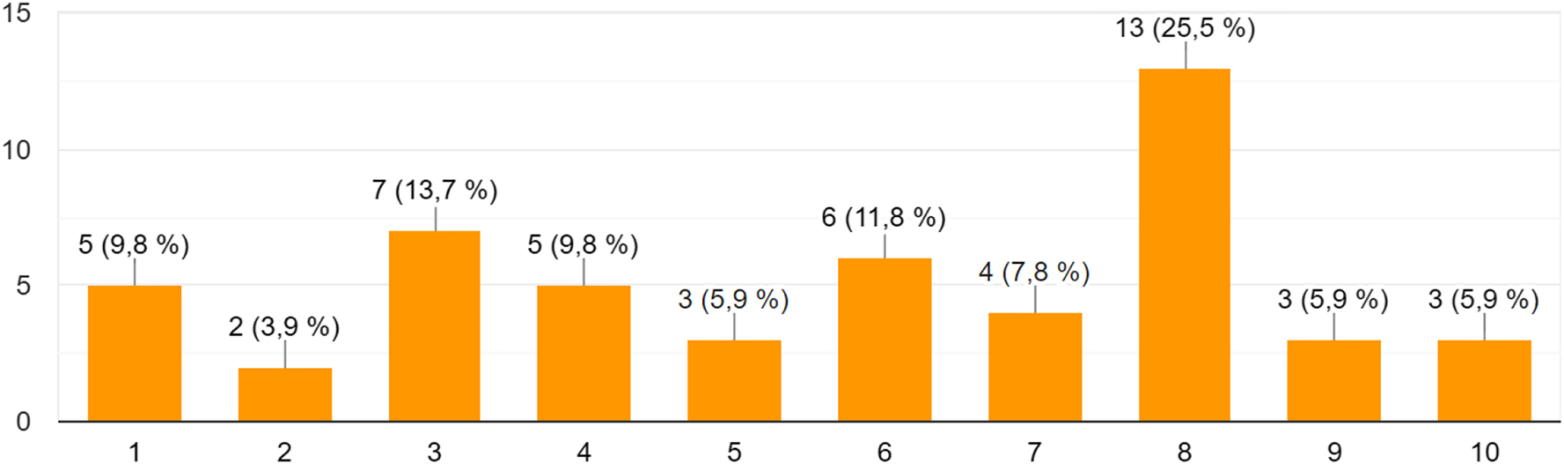
n=50 answers to the question: *As a remote participant, how much did you feel like a part of CARS 2020?* (1 = almost excluded – 10 = fully integrated).

A total of n=55 participants (which corresponds to roughly a quarter of all registered guests) responded to the questionnaire. Among those, 85% had been remote speakers or virtual guests. Most remarkably, the overwhelming majority of the audience (75%) had never attended a virtual conference before. Our key question was how much the participants felt themselves like a part of the conference. To express their subjective perception, a scale between 1 (“almost excluded”) and 10 (“fully integrated”) was provided (see Figure 7).

The answers were rather regularly distributed with a slight lead of the more positive answers.

**Figure 8:**
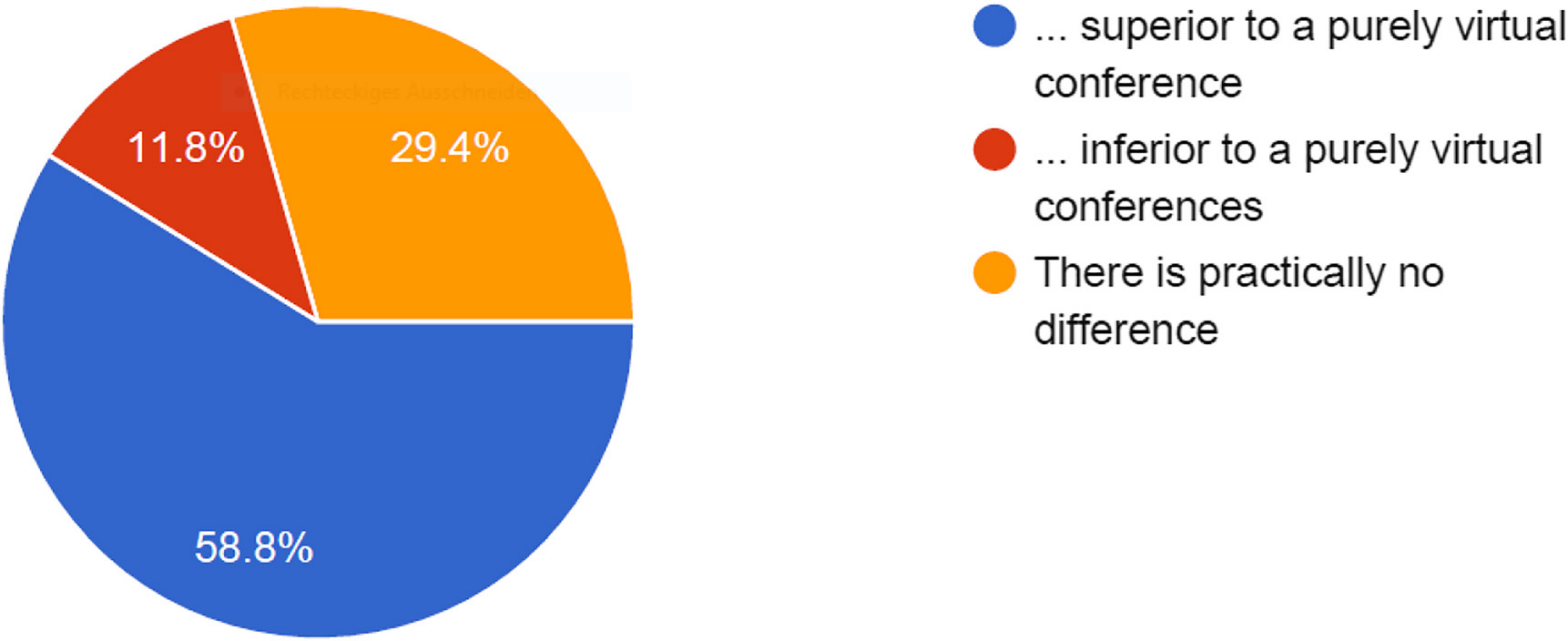
Responses to the question “*Do you think the hybrid format (local in-person + remote participation) is …?”.* Three different options were available. 60% of responders considered it as superior and 12% as inferior to purely virtual conferences.

Another question focused upon the personal recommendation of the participants in regards to further scientific congresses or similar meetings (see Figure 8).

## Discussion

Most scientific associations in 2020 had to struggle with a dramatic change: Regular, traditional meetings with personal communication and exchange, networking, and creation of new visions became obsolete almost instantly. As an alternative, virtual conferences became increasingly popular.

Beyond making conferences possible at all, virtual conferences even have some specific advantages compared to traditional on-site meetings: Since expenses for traveling and lodging are avoided, most participants save a significant amount of money, not to mention precious time which, otherwise, has to be spent during transit. On the other hand, a purely virtual conference is always a more or less “abstract” event. There is no way to have a short communication on a coffee with a friend or scientific partner. Receptions, dinners, and coffee breaks which are frequently a source of new scientific inspiration or the start of networks, do not exist in a way we are accustomed to. Modern systems offer to some extent surrogates like chatrooms, virtual villages, or even social events, but this needs the respective knowledge (and not of all the participants are digital natives) and, above all, very careful planning by both the organizers and the participants.

Searching for a viable alternative to purely virtual conferences, we shaped the format of a “hybrid” conference. At the time of planning, little has been known about how to organize bigger scientific teleconferences. The ACM [5] had published valuable guidelines, but they did not perfectly match our needs and ideas. Systematic descriptions such as by Rubinger [2] did appear only later. We tried to create a physical “center” of the meeting, although most of the scientific contributions would be virtual since we believe that location is always important to keep the event in memory. Every single annual meeting of the CARS has its own face: When we recall 2019, we think of Rennes and the lectures and discussions in the charming monastery. Similarly, we connect Fukushima – just to mention another example – with places, events, discussions, and new insights of 2017. The event, the time, the place, and foremost the people always belong together.

In a purely virtual conference, time is the only reference point. Mentally, we do not associate it with any country, town, or lecture hall. We aimed to connect the event with a location and to create something like a *déja vu* effect over the three days of the congress.

As an anchor point, we defined the lecture hall D in *Klinikum rechts der Isar*, Technical University of Munich, for pragmatic reasons. Though not particularly attractive from its outer appearance, it offered sufficient space to host several <50 persons per session without appearing too empty. It offered the full range of technological preconditions necessary for advanced telecommunication and was located in a surrounding allowing coffee breaks, dinner, etc. in the open air.

Certainly, those with the privilege to take part personally on-site did benefit most from the hybrid format. Those who were sitting in the rows were at least as close to the remote speaker as if he/she would be a speaker in a real, large conference hall.

For remote speakers, a look at a real audience was possible. As many speakers emphasized afterward, viewing a real although a small group of guests was stimulating and made them feel like being a part of a real meeting.

The remote spectators did not only see the individual speakers but also the podium of the chairpersons, and the audience as well. Insofar, this mixture of real and virtual elements should significantly improve the immersion.

The additional element of a real place augmented the organizational tasks considerably: “Organization replaces location”. This is why we established the technical chairs. Though soft – and hardware worked reliably, most chairpersons were continuously busy during the sessions. However, their activities were not mainly of technical nature but rather organizational ones. The CO’s took over to establish connections, to tune the voices, etc. The TC’s had to coordinate the interaction of the remote chairpersons, define the various camera settings, and serve as an eye for the remote chairs since they were the only ones who did always see all actors (chairpersons, speaker, debaters, and audience) simultaneously either directly or on one of the numerous screens in front of them.

The final takeaway question is if this immense effort justifies the hybrid format. The nasty problem of the time zone difference which is typical of international VC’s cannot be abolished by a hybrid conference, either. The only advantage is that time and event are now associated with a distinct physical location.

Even though the majority of those participants who responded to the questionnaire did not have personal experience with virtual conferences (and, thus, the knowledge of so many specific shortcomings of VC), most of them judged positively. We assume – but of course, cannot prove it – that the degree of acceptance would have been lower if CARS would have been purely virtual. On the other hand, the percentage of responders who found the hybrid format superior probably would have been higher if they had had experience with purely virtual conferences beforehand.

Many organizers of scientific meetings were confident at the end of the first infection wave of Covid-19 that classical conferences would soon become the standard again since there is no doubt at all, that they offer the maximum of formal and informal human interaction and exchange. However, after the outbreak of the second wave, teleconferencing lost its substitutional character and is being established as an option of its own right. For further practical implementation, our own – involuntary – early experience with a “mixed” real and virtual conference could be helpful.

## Supplementary Material

Supplementary Material
