# Supplementary material for: “Hybrid” scientific conference: lessons learned from the digital annual meeting of the CARS international conference during the Covid-19 pandemic
Source: Innov Surg Sci. 2021 Nov 9;6(3):115–23. doi: 10.1515/iss-2021-0012 (PMC8826162; doi:10.1515/iss-2021-0012)
Supplement: Supplementary file 1 — Supplementary Material [file j_iss-2021-0012_suppl.pdf]

Reviewer Assessment

D Ostler et al.: „Hybrid“ scientific conference: Lessons learned from the digital annual meeting of the CARS international conference during the Covid-19 pandemic

Reviewers’ Comments to Original Submission

Reviewer 1: anonymous

Date received: 06-April-2021
Reviewer recommendation: **Reject for the following reasons**
Reviewer overall scoring: **Unacceptable**

Assessment Form scores: 5 = High/Yes; 3 = Medium/Adequate; 1 = Low

Is the subject area appropriate for the journal					1
Does the title clearly reflect the paper's content?	5				
Does the abstract clearly reflect the paper's content		4			
Do the keywords clearly reflect the paper's content?		4			
Does the introduction present the problem clearly?	5				
Are the results/ conclusions justified?		4			
How comprehensive and up-to-date is the subject matter presented?	5				
How adequate is the data presentation?			3		
Are units and terminology used correctly?		4			
Is the number of cases adequate?			3		
Are the experimental methods/ clinical studies adequate?					
Is the length appropriate in relation to the content?			3		
Does the reader get new insights from the article?		4			
Please rate the practical significance.			3		
Please rate the accuracy of methods.			3		
Please rate the statistical evaluation and quality control.					1
Please rate the appropriateness of the figures and tables.		4			
Please rate the appropriateness of the references.		4			
Please evaluate the writing style and use of language.		4			
Please judge the overall scientific quality of the manuscript.				2	
Are the methods used worthy of reproduction in greater deal?	No				
Would you be willing to review a revision of this manuscript?	No				

Comments to author: This is an interesting paper, but I don't think it should be published in a scientific surgical journal. Please look out for a journal for medical information.

Comments by the Editor-in-Chief to the Submission

Dear Readers,
although scientific meetings are more and more online due to the corona pandemic situation, it was extremely difficult to find suitable reviewers for this interesting paper, which evaluated a digital international conference. We asked eight potential reviewer within Europe and the US, but nobody was able to review the manuscript. Therefore it was decided to publish this contribution although we had one reviewer who rejected the paper. Once again, we invite you to comment on that paper.
With best regards,
Prof. Joachim Jähne, EiC
